# Soil fungal communities varied across aspects of restored grassland in former mining areas of the Qinghai-Tibet Plateau

**DOI:** 10.1371/journal.pone.0295019

**Published:** 2024-03-26

**Authors:** Xiaoqing Li, Qiang Li, Yinzhu Duan, Haiqun Sun, Hui Chu, Shunbin Jia, Hongjie Chen, Wenxi Tang

**Affiliations:** 1 College of Agriculture and Animal Husbandry, Qinghai University, Xining, China; 2 Qinghai Province Grassland Improvement Experiment Station, Xining, China; 3 Qinghai Province Grassland Technology and Extension Station, Xining, China; 4 Tongyu County Animal Quarantine Station, Baicheng, China; Friedrich Schiller University, GERMANY

## Abstract

To determine whether different aspects lead to a heterogeneous distribution of soil fungi, we investigated artificially established alpine grasslands in the Muli mining area in the Qinghai-Tibet Plateau. Employing high-throughput sequencing techniques, we analyzed the composition, diversity, and function of soil fungal communities across various aspects (flat, East-facing, South-facing, West-facing, North-facing). We also examined their relationships with environmental factors. Soil fungal communities of restored alpine grasslands differed significantly across aspects in terms of the dominant phyla, classes and species level. Compared with No aspect, the Shannon index of fungi respectively decreased by 2.99%, 19.32%, 19.37% and 10.56% for East aspect, South aspect, West aspect and North aspect, respectively, and the Chao1 index of fungi respectively decreased by-2.44%, 35.50%, 42.15% and 3.21%, respectively. A total of 22 different types of fungi were identified in the study area. Predictive analysis, based on PICRUSt2, indicated that the primary functions of the fungal communities across different aspects were aerobic respiration I (cytochrome c) and aerobic respiration II (cytochrome c). Among the environmental variables, total phosphorus (P) and total nitrogen (N) were the principal factors influencing the fungal community composition.In conclusion, aspect plays a significant role in shaping the composition of fungal communities and also affects their overall diversity.

## Introduction

The microorganism community is an important component of belowground biodiversity and plays an important role in ecosystem mass cycling and energy flow [1]. Soil fungi are one of the most important soil microorganisms which may decompose complex organic matter and form parasitic or symbiotic relationships with plants [2]. They hence play an important role in decomposing organic matters, nutrient cycling [3], and maintaining soil ecosystem stability and biodiversity [4]. There is a clear functional differentiation in soil fungi. For example, saprophytic fungi are important decomposers of soil organic matter; they obtain energy from decomposing organic matter and altered the rate of carbon and other mass cycling in the ecosystem [5]. Mycorrhizal fungi formed a symbiosis relationship with plants, where plants provide carbon hydrates for mycorrhizal fungi and mycorrhizal fungi provide nutrients like nitrogen and phosphorus in return [6]. Plant pathogenic fungi infected plant tissues and impede plant growth and reproduction [7]. In addition, fungi mycelia form a complex soil food web together with other soil organisms [8]. The diversity and community structure of fungi can reflect soil nutrients and soil health, which serves as one of the important indicators for evaluating soil health [9]. Distribution patterns of soil fungi are environment-dependent. When external environmental conditions (soil physicochemical properties, vegetation types and climatic conditions etc.) change, tructure and function of fungal communities would change accordingly [10–12]. It is hence important for soil management and ecosystem maintenance purpose to have an in-depth understanding of soil fungal diversity, the characteristics of different functional groups composition, and their influence mechanisms.

Topography plays a crucial role in shaping mountain ecosystems by contributing to their spatial and temporal heterogeneity [13]. At medium and large scales, topography redistributes water and heat over time and space through elevation and slope aspects, resulting in substantial variations in regional microclimates. At a local scale, differences in radiation, light, wind speed, and precipitation are primarily influenced by the orientation of slope aspects. In other words, slope aspects are the main environmental factor determining vegetation distribution, soil nutrient levels, and microorganism presence in montane ecosystems, whether at a meso-upper or local scale [14–16]. As aspects influence the amount of solar radiation received by the ground and the angle at which prevailing winds intersect with the surface, environmental factors at slopes change accordingly, such as light, heat, water, and soil minerals [17]. These differences in turn create microclimatic gradients across various slope aspects, ultimately influencing the spatial distribution patterns of different biomes. While current study focuses mainly on the impact of slope aspects on plant taxa, there is limited investigation into soil microbial taxa, particularly fungal communities, in alpine grasslands. Consequently, it is essential to analyze the structure and diversity of soil fungal communities across different slope aspects to understand their contribution to vegetation recovery and response to environmental changes in mountain ecosystems.

The Muli region of Qinghai Province, China, is located at the mid-eastern part of the Qilian Mountains on the Qinghai-Tibet Plateau [18] adjacent to the Qilian Mountains National Park. It serves as an essential ecological security barrier and genetic gene pool for species in northwest China, which makes it a priority area for biodiversity conservation and water conservation [19]. However, open-pit mining activities in Muli area in recent years have caused many coal gangue mountains around mining sites, causing vegetation destruction and soil erosion, which seriously threatened regional ecological and environmental security [20, 21]. A grassland restoration project initiated in 2013 with reconstituted soil and replanted grass seeds [20]. This project successfully restored the open mine pit into grass land. At present, studies on ecological restoration in the Muli region have revealed the changes in plants and soil properties after the restoreation [18–22], but the soil fungi have not been reported. Moreover, because all restoration areas applied similar reconstituted soil and replanted grass species, such hence provided an unique opportunity to study the effect of aspects on the distribution pattern of soil fungal communities. The results may help to understand the construction mechanism of fungal communities at local scales across different aspect.

## Materials and methods

### Study area

The study area is located in the Juhugeng coal mine within the Muli coalfield in the northeast edge of the Qinghai-Tibet Plateau, which is located in the Tianjun County, Qinghai Province, (99°27′-99°35′E, 38°02′-38°03′N). The Muli mining area covers an area of about 55 km^2^ with an average altitude of ~3800 m. The annual average temperature is -5. 0°C, and the minimum temperature can reach -36. 0°C; the annual average precipitation is > 500 mm. This area has a typical plateau mountain climate with cold and dry winter and cool and humid summer.. The soil types of the original community around the study area were mainly swamp soil and alpine meadow soil, and the main native plant species were *Koeleria tibetica* and *Carex moorcroftii*.

Grass restoration initiated in 2019 in the former mining area with a seeding rate of 300 kg hm^-2^. Planted grass species included *Elymus nutans*, *Poa crymophila*, and *Puccinellia tenuiflora*, which were sown at a ratio of 2:1:1. The soil was reconstituted soil with a thickness of 30cm, mainly composed of muck, sheep slab manure, and organic fertilizer. Soil nutrient background value at the time of the restoration in 2013 was total N 1.78 g·kg^-1^, total P 0.85 g·kg^-1^, total K 22.11 g·kg^-1^, available N 16.50 mg·kg^-1^, available P 1.93 mg·kg^-1^, available K 70.75 mg·kg^-1^, soil organic matter 59.89 g·kg^-1^, and soil pH 8.63.

### Experimental design and sample collection

In August 9^th^, 2021, five different aspects (No aspect, East aspect, South aspect, West aspect, North aspect) were selected in the Juhugeng coal mine area, and the *No aspect* area was used as the control. Three sampling areas of 10 × 10 m were selected for each aspect (Table 1). Meanwhile, the soil samples were collected with permission from the Muli region of Qinghai Province Administration, Qinghai province, China. All authors committed that all methods were carried out in accordance with relevant guidelines and regulations.

**Table 1:** Basic information of sample plots at different aspect. All plots shared three dominant species, *Elymus nutans*, *Poacrymophila and Puccinellia tenuiflora*.

Aspects	Altitude m	Latitude	Longitude	Coverage %
No aspect	3908	E99º11′55″	N38º06′43″	88–90
East aspect	4133	E99°04′49"	N38°08′26"	79–81
South aspect	4070	E99°08′43″	N38°06′28″	83–85
West aspect	4074	E99°09′51 "	N38°07′51 "	89–91
North aspect	4045	E 99°07′05″	N 38°08′47″	94–96

Three 0.5× 0.5 m quadrats were established inside each sampling area. After the grass samples were cut, the surface soil samples of 0–20 cm were collected, and three soil samples were taken in each quadrat. The first sample was taken with a ring knife to measure soil water content, the second was used for subsequent determination of soil indicators, and the third was used to determine soil fungal communities.

### Indicator determination

#### 1) Soil properties

Soil water content were measured by the weight difference between fresh soil samples and oven-dried samples. Soil pH and conductivity were measured by a water-soluble acidity meter and conductivity meter. Soil organic carbon (SOC) was determined by an automatic carbon analyzer (Multi N/C 2100S/1, Analytik Jena AG, Germany). Soil total nitrogen was determined by the Kjeldahl method. Soil available N was determined by the alkaline diffusion method. Soil available P was determined by the molybdenum antimony anti-colorimetric method Measured. Soil available K was measured by the ammonium acetate-flame photometer method.

#### 2) Fungi DNA extraction, qPCR and Illumina MiSeq sequencing

Total DNA in soil samples were extracted using the E.Z.N.A.^®^ soil DNA kit (Omega Bio-tek, USA). DNA purity and concentration were measured by 1% agarose gel electrophoresis and absorbance values at 260/280 nm and 260/230 nm with a Nanodrop^®^ ND-2000 UV spectrophotometer (NanoDrop Technologies, USA). The extracted DNA was diluted and stored in TE buffer (10 mmol L^-1^ Tris-HCl, 1 mmol L^-1^ EDTA, pH was 8.0) and kept at -20°C for further use.

The relative abundance of fungal ITS genes was determined using a StepOne real-time fluorescence PCR instrument (ABI 7500, Applied Biosystems, USA) with amplification primers ITS1 (5′-CTTGGTCATTTAGAGGAAGTAA-3′) and ITS2 (5′-TGCGTTCTTCATCGATGC-3′), respectively. The 20 μL reaction system consisted of 10 μL of highly sensitive dye-quantitative PCR detection reagent (Vazyme Biotech Co., Ltd, China), 0.8 μL each of forward and reverse primers (5 μmol-L-1), 1 μL of DNA template, and 7.4 μL of plasma water. The amplification conditions were pre-denaturation at 95°C for 5 min, running 40 cycles at 95°C for 30 s per each cycle, annealing at 58°C for 30 s, and extension at 72°C for 1 min. PCR products were recovered on 2% agarose gels and AxyPrep DNA gel kits (Axygen iosciences, USA) and quantified with QuantiFluor^™^-ST microfluorescents (Promega Corporation, USA). The purified PCR products were mixed and sequenced from both ends on the Illumina MiSeq high-throughput sequencing platform (Illumina, USA), and the sequencing was carried out by Shenzhen Microcomputer Technology Group Co., LTD.

### Data processing and analysis

We used SPSS21.0 for one-way ANOVA and interaction analysis. Soil fungal community composition, community diversity, LEfSe analysis and PICRUSt2 function prediction were completed using the Wekemo Bioincloud (https://www.bioincloud.tech) We performed redundancy discriminate analysis (RDA) with Monte Carlo permutation test for XXX data using Canoco 5.0 software (Microcomputer Power, Ithaca, USA). Error bars are shown as mean ± standard error.

## Results

### Soil properties

**Fig 1:**
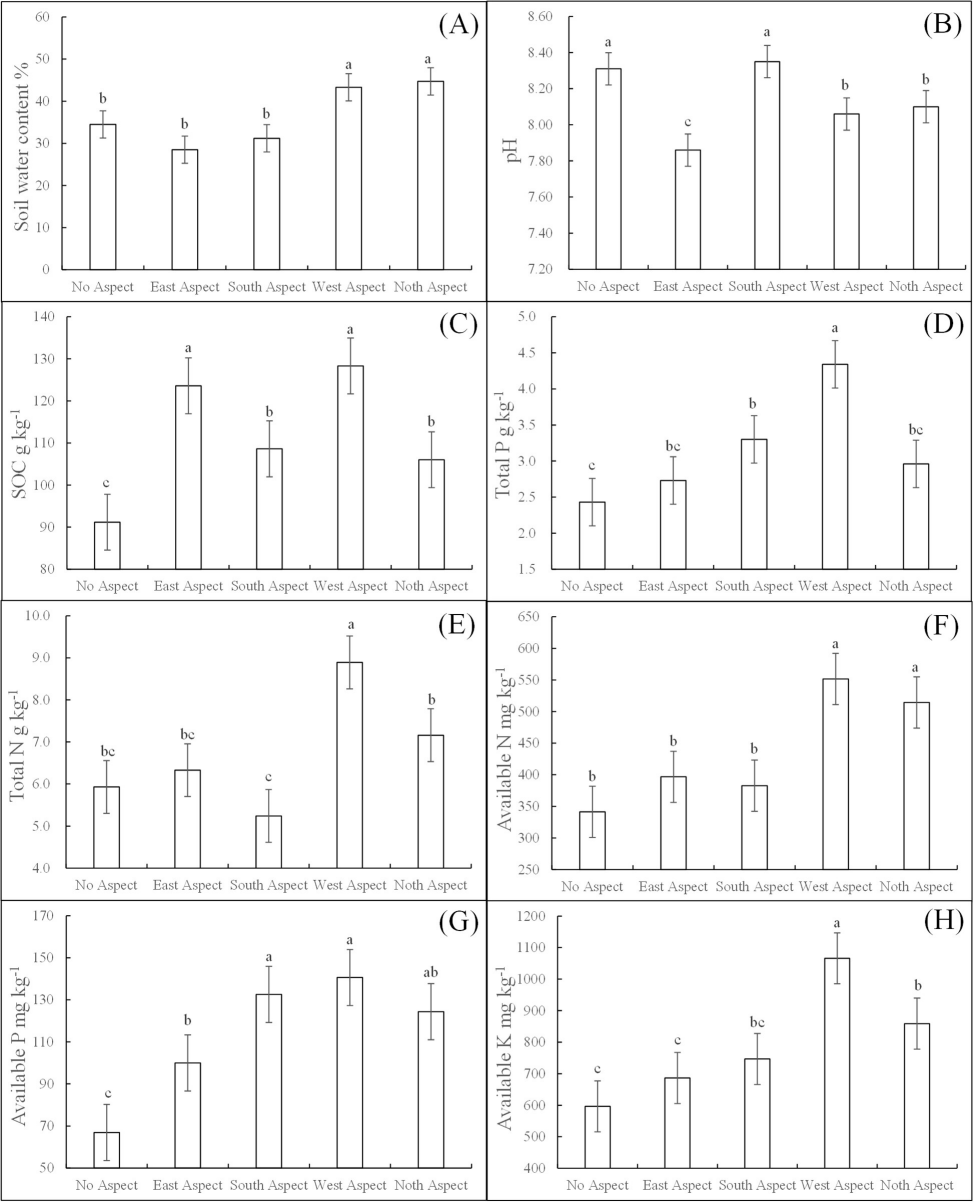
Soil properties of different aspects in the restoration area. SOC and available P were significantly lower at No Aspect than all other slopes.

Aspect exerted a significant influence on several soil properties, including water content, pH, SOC (Soil Organic Carbon), Total N, Total P, available N, available P, and alkaline K, as illustrated in Fig 1. As the aspect transitioned from No aspect, through East, South, West, and culminating in North, both Total P, available N, available P, and alkaline K exhibited an initial increase followed by a decrease. Conversely, soil water content showed an initial decline, followed by a rise. The sequence for soil water content across different aspects was: North aspect > West aspect > No aspect > South aspect > East aspect (Fig 1A). For pH, the order was: South aspect > No aspect > North aspect > West aspect > East aspect (Fig 1B). The order for SOC was: West aspect > East aspect > South aspect > North aspect > No aspect (Fig 1C), and for total N it was: West aspect > North aspect > East aspect > No aspect > South aspect (Fig 1E). For total P, available P, and alkaline K, the sequence was: West aspect > North aspect > South aspect > East aspect > No aspect (Fig 1D, 1G and 1H). Lastly, available N ranked as: West aspect > North aspect > East aspect > South aspect > No aspect (Fig 1F). Compared to the No aspect condition: Soil water content demonstrated an increase of -17.39% for East aspect, -9.57% for South aspect, 25.51% for West aspect, and 29.57% for North aspect. PH values registered changes of -5.42% for East aspect, 0.48% for South aspect, -3.01% for West aspect, and -2.53% for North aspect. SOC exhibited increases of 35.54% for East aspect, 19.12% for South aspect, 40.73% for West aspect, and 16.26% for North aspect.Total N showed variations of 6.75% for East aspect, -11.64% for South aspect, 49.92% for West aspect, and 20.74% for North aspect. Total P demonstrated increases of 12.35% for East aspect, 35.80% for South aspect, 78.60% for West aspect, and 21.81% for North aspect.Available N presented increases of 16.21% for East aspect, 12.11% for South aspect, 61.62% for West aspect, and 50.68% for North aspect. Available P showcased increases of 49.37% for East aspect, 98.01% for South aspect, 110.03% for West aspect, and 85.76% for North aspect. Alkaline K displayed increases of 15.09% for East aspect, 25.21% for South aspect, 78.76% for West aspect, and 43.99% for North aspect.

### Soil fungal community composition

**Fig 2:**
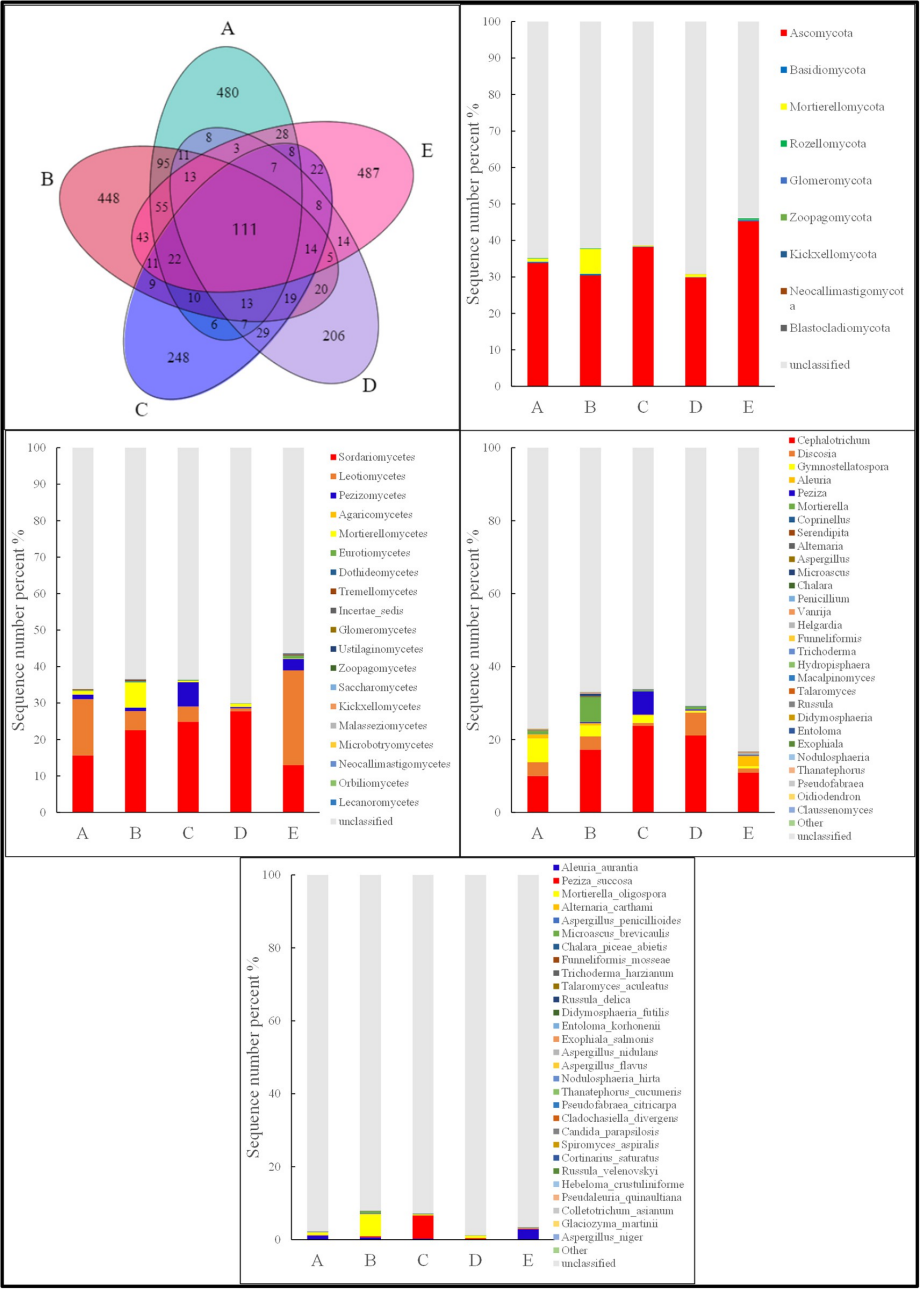
Soil fungal community composition of different aspects in the restoration area. Note: A: No aspect; B: Ease aspect; C: South aspect; D: West aspect; E: North aspect.

In the artificial planting of alpine grassland across different aspects, the common Operational Taxonomic Units (OTUs) of soil fungal communities was 111 (Fig 2A). The unique OTUs of soil fungal communities were ranked as follows: North aspect (487) > No aspect (480) > East aspect (448) > South aspect (248) > West aspect (206).

At the phylum level (Fig 2B), the dominant phyla in the soil fungal communities of the artificially planted alpine grassland included Ascomycota, Basidiomycota, and Mortierellomycota, with respective abundances of 35.53%, 0.25%, and 1.78%. Ascomycota was particularly prominent. The relative abundance of fungi across different phyla exhibited significant variation with aspect changes (P<0.05). The order of relative abundance for Ascomycota was North aspect > South aspect > No aspect > East aspect > West aspect (Fig 2B). Compared to the No aspect condition, the relative abundance of Ascomycota changed by -10.05% for East aspect, 12.91% for South aspect, -11.75% for West aspect, and 33.80% for North aspect.

At the class level (Fig 2C), dominant classes within the soil fungal communities were *Sordariomycetes*, *Leotiomycetes*, *and Pezizomycetes*, with abundances of 20.75%, 10.32%, and 2.46% respectively. *Sordariomycetes* was especially prominent. The relative abundance of fungi varied significantly among the classes with the shift in aspects (P<0.05). The abundance ranking for *Sordariomycetes* was West aspect > South aspect > East aspect > No aspect > North aspect; for *Leotiomycetes*, it was North aspect > No aspect > East aspect > South aspect > West aspect; and for *Pezizomycetes*, it was South aspect > North aspect > No aspect > East aspect > West aspect.

At the genus level (Fig 2D), dominant genera in the soil fungal communities included *Cephalotrichum*, *Discosia*, and *Gymnostellatospora*, with abundances of 16.59%, 3.13%, and 2.53%, respectively, with Cephalotrichum being particularly notable. The relative abundance of fungi among different genera showed significant variation with aspect changes (P<0.05).

At the species level (Fig 2E), *Aleuria_aurantia* was the dominant species in both No aspect and North aspect. *Mortierella_oligospora* was dominant in the East aspect. *Peziza_succosa* was the dominant species in the South aspect. In the West aspect, both *Peziza_succosa* and *Mortierella_oligospora* were dominant.

### Soil fungal community diversity

**Fig 3:**
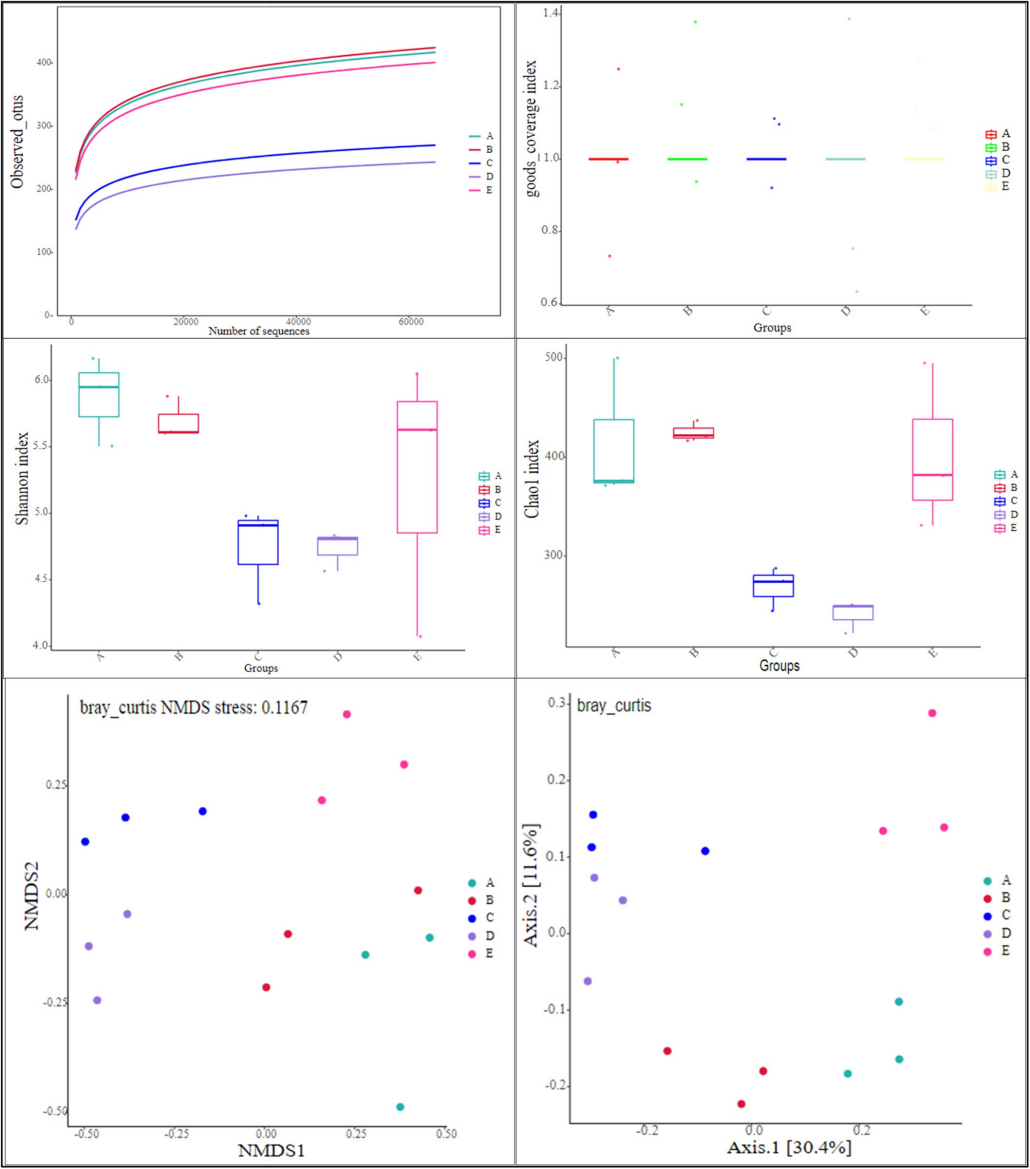
Soil fungal community diversity of different aspects the restoration area. Note: A: No aspect; B: Ease aspect; C: South aspect; D: West aspect; E: North aspect.

It is evident from Fig 3A that the number of fungal OTUs in the dilution curve shifts gradually with the increase in sequencing volume, indicating that the soil samples from artificially planted alpine grassland across different aspects were adequately sequenced. The goods coverage values for different aspects all exceeded 0.995 (Fig 3B), suggesting that the sequencing depth effectively captured the variations in the soil fungal community of the artificially planted alpine grassland.

The ranking for the Shannon index of fungi was as follows: No aspect > North aspect > East aspect > South aspect > West aspect (Fig 3C). When compared to the No aspect condition, the Shannon index for fungi decreased by 2.99% for the East aspect, 19.32% for the South aspect, 19.37% for the West aspect, and 10.56% for the North aspect. The ranking for the Chao1 index of fungi was: East aspect > No aspect > North aspect > South aspect > West aspect (Fig 3D). In comparison to the No aspect condition, the Chao1 index for fungi decreased by 2.44% for the East aspect, 35.50% for the South aspect, 42.15% for the West aspect, and 3.21% for the North aspect.

Based on NMDS and PCoA analyses, significant differences were observed in the β-diversity of soil fungal communities across different aspects (P<0.05) (Fig 2E and 2F). This means that the fungal communities from different aspects can be clearly differentiated and exhibit significant differences.

### Soil fungi LEfSe analysis and PICRUSt2 function prediction

**Fig 4:**
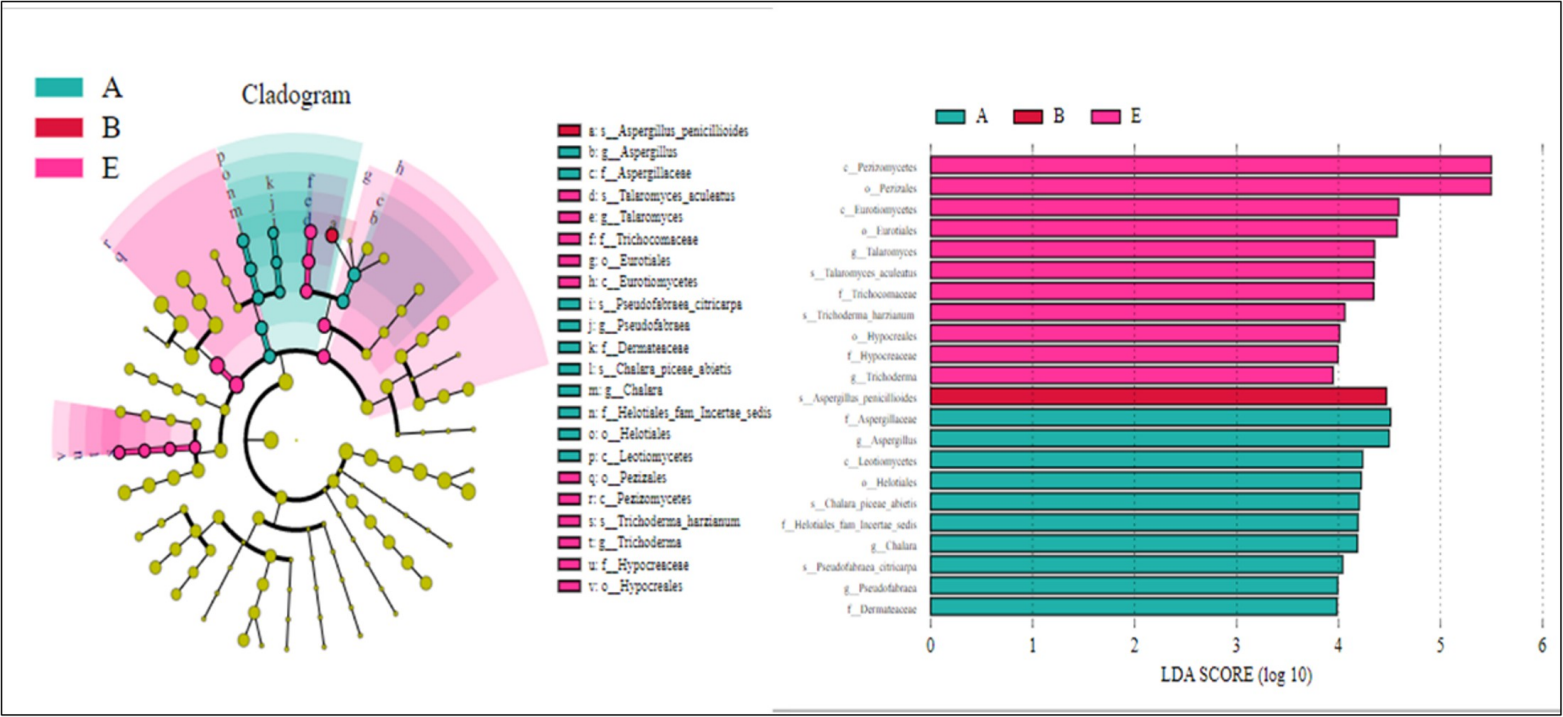
LEfSe analysis of different aspects in revegetation area.

From Fig 4, setting an LDA threshold of 3.0 identified 22 species with variations at different taxonomic levels for fungi across various aspects. The number of distinct soil fungal species across different aspects differed significantly (P < 0.05). Specifically, the species varying across different taxonomic levels for No aspect, East aspect, and North aspect were 10, 1, and 11, respectively.

**Fig 5:**
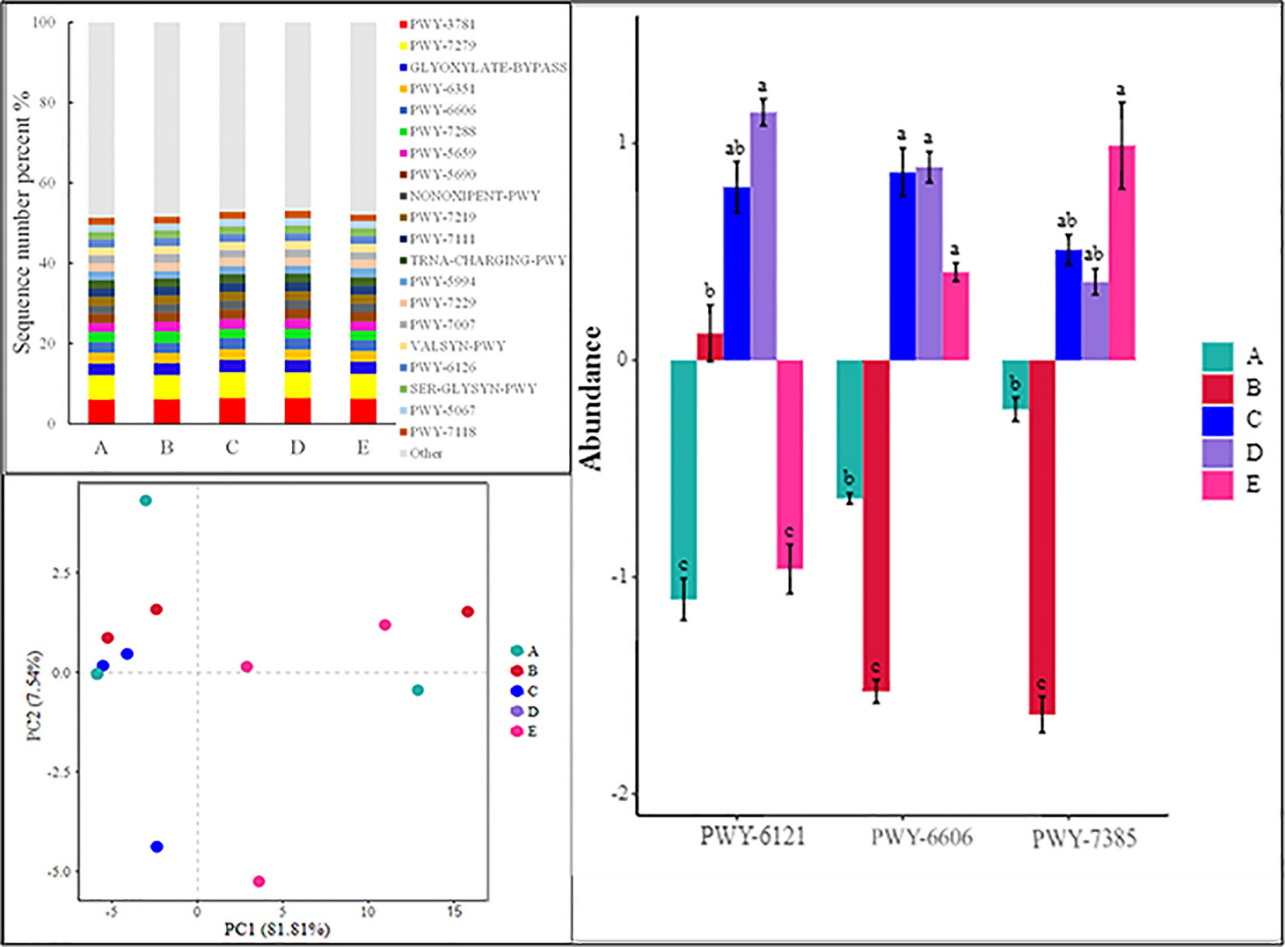
PICRUSt2 function prediction of different aspects in revegetation area. Note: GLYOXYLATE-BYPASS: glyoxylate cycle; NONOXIPENT-PWY: pentose phosphate pathway (non-oxidative branch); PWY-3781: aerobic respiration I (cytochrome c); PWY-5067: glycogen biosynthesis II (from UDP-D-Glucose); PWY-5659: GDP-mannose biosynthesis; PWY-5690: TCA cycle II (plants and fungi); PWY-5994: palmitate biosynthesis I (animals and fungi); PWY-6126: superpathway of adenosine nucleotides de novo biosynthesis II; PWY-6351: D-myo-inositol (1,4,5)-trisphosphate biosynthesis; PWY-6606: guanosine nucleotides degradation II; PWY-7007: methyl ketone biosynthesis; PWY-7111: pyruvate fermentation to isobutanol (engineered); PWY-7118: chitin degradation to ethanol; PWY-7219: adenosine ribonucleotides de novo biosynthesis; PWY-7229: superpathway of adenosine nucleotides de novo biosynthesis I; PWY-7279: aerobic respiration II (cytochrome c) (yeast); PWY-7288: fatty acid &beta;-oxidation (peroxisome, yeast); SER-GLYSYN-PWY: superpathway of L-serine and glycine biosynthesis I; TRNA-CHARGING-PWY: tRNA charging; VALSYN-PWY: L-valine biosynthesis.

PICRUSt2 classifies species based on fungi, allowing the extraction of the ecological functions corresponding to these fungi. As shown in Fig 5, the proportion of soil fungi annotated with functions across different aspects ranged between 51.36% and 53.09%, all exceeding 50% (Fig 5A). Excluding the unknown flora, soil fungi in the artificially planted alpine grassland across different aspects encompassed ecological functional groups such as the glyoxylate cycle, pentose phosphate pathway (non-oxidative branch), aerobic respiration I (cytochrome c), glycogen biosynthesis II (from UDP-D-Glucose), GDP-mannose biosynthesis, and TCA cycle II (plants and fungi). Other notable pathways included palmitate biosynthesis I (animals and fungi), superpathway of adenosine nucleotides de novo biosynthesis II, and L-valine biosynthesis. Noteworthy among these are the aerobic respiration I (cytochrome c) and aerobic respiration II (cytochrome c) (yeast). PCA analysis (Fig 5B) demonstrated that the functional traits of soil fungi could be clearly differentiated. Concurrently, specific pathways such as the superpathway of adenosine nucleotides de novo biosynthesis II, guanosine nucleotides degradation II, and 1,3-propanediol biosynthesis (engineered) exhibited significant differences across different slopes (Fig 5C).

### Relationship between soil fungal community and environmental factors

**Fig 6:**
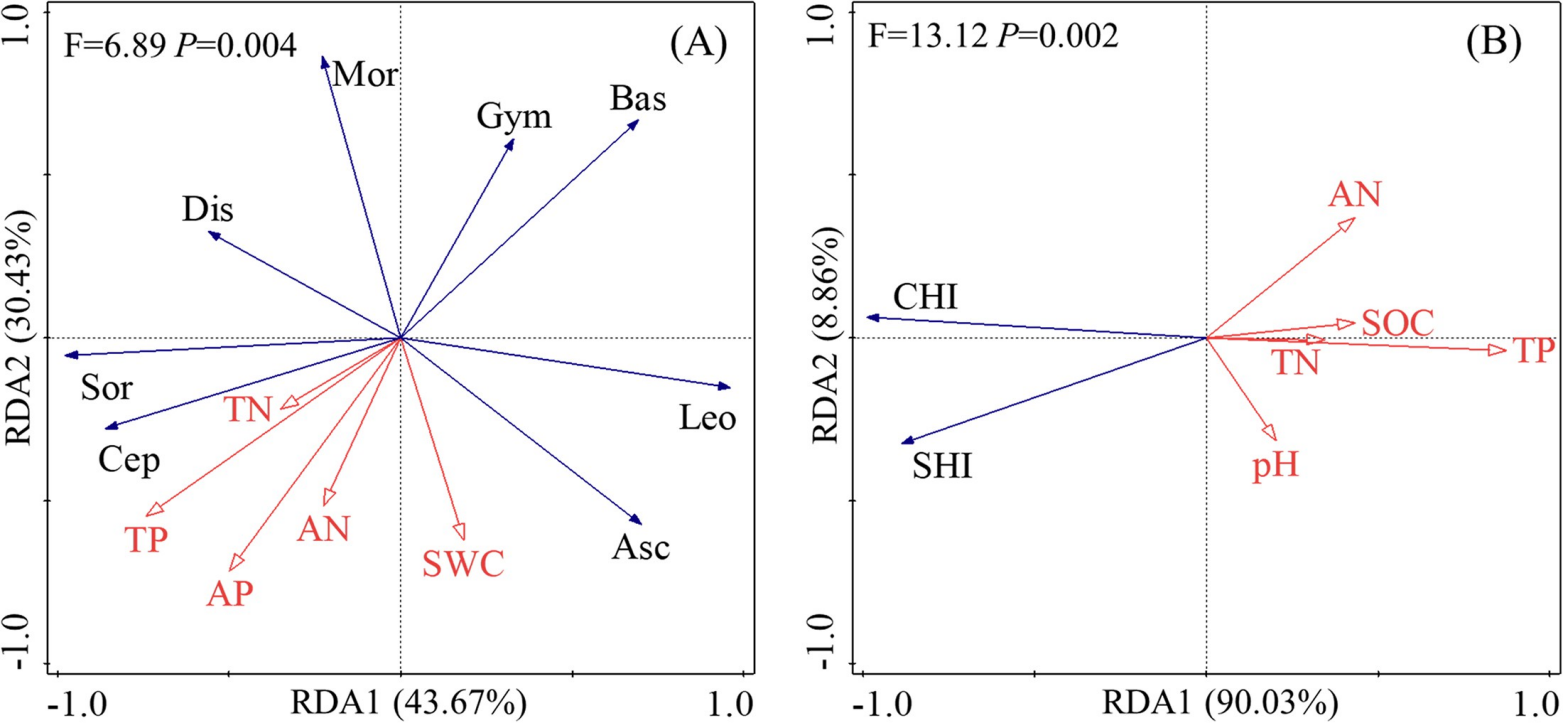
RDA analysis of environmental factors and fungal community composition (A), diversity (B). Note: TN: Total N; TP: Total P; AN: Available N; AP: Available P; AK: Available K; SWC: Soil water content; Asc: Ascomycota; Bas: Basidiomycota; Mor: Mortierellomycota; Sor: Sordariomycetes; Leo: Leotiomycetes; Cep: Cephalotrichum; Dis: Discosia; Gym: Gymnostellatospora; CHI: Chao1 index; SHI: Shannon index.

An RDA analysis was conducted to study the relationships between environmental factors and both the composition (relative abundance of dominant phylum, class, and genus) and diversity (Shannon index, Chao1 index) of the soil fungal community (Fig 6A and 6B, respectively). As shown in Fig 6, the first two axes of environmental factors explained 74.10% and 98.89% in soil fungal community composition and diversity, which are statistically significant. This indicates that these two axes effectively capture the relationship between soil fungal community composition, diversity, and the associated environmental factors. Based on the Monte Carlo test in the RDA analysis (Table 2), total P, soil water content, total N and available P significantly influenced fungal community composition (*P* < 0.01). Furthermore, total P and total N had a significant impact on fungal community diversity (P < 0.01). Collectively, this suggests that total P and total N are the primary environmental determinants of fungal community dynamics.

**Table 2:** Results of the redundancy discrimination analysis (RDA) through Monte Carlo permutation test of soil fungal community composition and diversity based on environmental factors.

Soil fungal community composition				Soil fungal community diversity			
Name	Contribution %	pseudo-F	P	Name	Contribution %	pseudo-F	P
TP	34.9	6.9	**0.002**	TP	74.6	33.5	**0.002**
SWC	27.2	8.5	**0.002**	TN	17.9	19.5	**0.002**
TN	22.3	15.2	**0.002**	SOC	2.5	3.2	0.074
AP	15.2	148	**0.002**	pH	3.4	6.5	0.018
AN	0.4	6.7	0.018	AN	1.7	4.3	0.028
SOC	0.3	<0.1	1	SWC	1.4	<0.1	1
pH	0.3	<0.1	1	AP	1.2	<0.1	1
AK	0.2	<0.1	1	AK	0.8	<0.1	1

Note: TN: Total N; TP: Total P; AN: Available N; AP: Available P; AK: Available K; SWC: Soil water content.

## Discussion

### Composition of soil fungal communities

Soil fungi play a crucial role in the decomposition of animal and plant residues, acting as essential drivers of the nitrogen and carbon cycles in soil. Particularly in the early stages of plant organism decomposition, fungi exhibit greater activity than bacteria and actinomycetes. In montane ecosystems, the terrain’s aspect introduces heterogeneity in environmental factors such as light, heat, water, and soil nutrients. This heterogeneity arises from variations in solar radiation received by the ground and the angle at which prevailing winds intersect with the terrain [23]. Such conditions lead to microclimatic variations across different aspects, subsequently affecting the composition and diversity of soil fungal communities [24]. This terrain aspect also influences soil’s physicochemical properties, notably temperature and water content. As a consequence, microclimatic variations across different aspects can introduce variations in the orientation and distribution of soil fungi [24].

Our study identified significant differences in soil water content and nutrient content across different aspects, findings that align with those of Sidari et al. [24]. Further, we found that the unique OTUs of soil fungal communities followed this order: North aspect > No aspect > East aspect > South aspect > West aspect. This suggests that different aspects tend to decrease the number of differential OTUs in fungal communities. Zhou et al. [25] reported that the most abundant phyla were *Actinobacteria* (39.90%) and Proteobacterial (39.90%), with the most prevalent classes being Actinobacteria (39.85%) and Acidobacteria (21.81%). In contrast, our research pinpointed Ascomycota as the dominant phylum (35.53%). Notably, the dominant classes were Leotiomycetes (10.32%) and Sordariomycetes (20.75%), while the dominant genus was Cephalotrichum (16.59%).

The disparities between our results and those of Zhou et al. [25] and Li et al. [26] regarding the composition of soil fungi in alpine grasslands can be attributed to the unique nature of our study area. Specifically, our study was conducted in an artificially established grassland in an alpine mining region, with the soil being reconstituted [27]. Although this grassland has been in place for 2 years, the evolution of the soil fungal community remains slow. As such, it has not yet achieved the compositional structure characteristic of the original alpine grassland soil fungal community. Within our study, Ascomycota was the dominant phylum (35.53%), with the abundances of Basidiomycota and Mortierellomycota being 0.25% and 1.78%, respectively. This dominance of Ascomycota can be attributed to their adaptability to alpine climates and resilience against alpine responses, making them more survivable than Basidiomycota and Mortierellomycota. Additionally, the high organic matter, nitrogen, and phosphorus content in the reconstituted soil provide a conducive environment for Ascomycota fungi to thrive, especially given that many of them are saprophytic and can break down various hard-to-degrade substances. These factors combined account for the observed variability in dominant fungal phyla, classes, and genera between our study and those of Zhou et al. [25] and Li et al. [26].

Our study also revealed significant variations in the relative abundance of dominant fungal phyla, classes, and genera across different aspects. For instance, the relative abundance of Ascomycota and Pezizomycetes in both the North and South aspects exceeded that in the No aspect. Conversely, their abundance in the East and West aspects was below the No aspect benchmark. Similarly, Sordariomycetes’ abundance in the West, South, and East aspects was higher than in the No aspect, but lower in the North aspect. This indicates that different aspects influence the relative abundance of dominant fungal phyla, classes, and genera to varying degrees. Such variability across fungal taxonomic levels results in certain aspects either increasing or decreasing the relative abundance of dominant fungal taxa when compared to the CK (No aspect) baseline. The underlying mechanisms causing these variations in fungal composition in our study area—an artificially established alpine grassland aimed at ecological restoration in a mining zone—warrant further, in-depth exploration.

### Diversity and functional prediction of soil fungi

Soil fungal α-diversity serves as a crucial indicator when evaluating the diversity of fungal communities and plays a pivotal role in ecosystem restoration and sustainability [12]. Generally speaking, the greater the diversity and complexity of soil microbial communities, the more stable the soil ecosystem becomes, making it more resistant to external disturbances [27]. In our research, compared with the No aspect, the Shannon index of fungi decreased by 2.99%, 19.32%, 19.37%, and 10.56% for the East, South, West, and North aspects, respectively. Similarly, the Chao1 index of fungi dropped by 2.44%, 35.50%, 42.15%, and 3.21%, respectively. Thus, it seems that aspect reduces soil fungal community diversity, potentially due to the combined effects of soil moisture and heat. Varying amounts of solar radiation received by different aspects lead to distinct soil temperatures. Concurrently, these radiation differences also result in diverse rates of soil evaporation. As a result, soil hydrothermal changes in alpine grasslands, triggered by aspect variations, collectively constrain the diversity of soil microorganisms [14–16]. Additionally, our NMDS and PCoA analysis revealed significant differences in the β-diversity of soil fungal communities across various aspects, indicating that aspects significantly influence the diversity of soil fungal communities.

LEfSe analysis demonstrated that the species at different taxonomic levels of soil fungal communities for No aspect, East aspect, and North aspect were 10, 1, and 11, respectively. This suggests that the West and South aspects might not exhibit species differentiation. The LEfSe analysis results further support the idea that aspect diminishes soil fungal diversity. Previous studies have posited that soil fungi exhibit a more intricate life history compared to bacteria. Some fungi have even evolved various nutritional strategies to adapt to fluctuating environments, signifying a sophisticated survival approach that enables them to adjust to diverse living conditions [22]. Our study discovered that the predicted function of the fungal communities, across different aspects, mainly focused on aerobic respiration I (cytochrome c) and aerobic respiration II (cytochrome c), based on PICRUSt2 analysis. Given that the soil in our study area is reconstituted, with a thickness of 30 cm and primarily comprising muck, sheep slab manure, and organic fertilizer—and is rich in carbon, nitrogen, and phosphorus—it stands to reason that the primary functions of fungi in this context are aerobic respiration I (cytochrome c) and aerobic respiration II (cytochrome c).

### Main environmental factors affecting soil fungal communities

In grassland ecosystems, soil fungi, as crucial members of soil microorganisms, serve as intermediary links between above-ground plants and below-ground soil. They actively participate in ecosystem processes such as plant apoplastic decomposition, nutrient cycling, and root nutrient uptake. Their role significantly affects plant growth, competition, ecosystem function, and stability in specific topographic microenvironments [23]. Within the alpine mountain ecosystem, different aspects can exhibit variations in soil ammonium nitrogen, nitrate nitrogen, plant, and available ecological stoichiometric ratio characterizations [28–31]. These differences inevitably influence the composition and diversity of soil fungal communities [32]. Hence, understanding the factors that influence the composition and diversity of soil fungal communities is pivotal for enhancing the balance and stability of ecosystems [8].

Zhu et al. [10] determined that factors such as soil water content, total N, SOC, and available N were more influential on fungal communities. Similarly, Ren et al. [33] identified that soil organic matter, total N, available N, available P, available K, and water content were intimately connected with alterations in fungal community structure. In our research, the Monte Carlo test in the RDA analysis revealed that total P, soil water content, total N, and available P significantly affected fungal community composition; whereas total P and total N had a pronounced impact on fungal community diversity (P < 0.01). In summary, total P and total N emerged as primary environmental factors influencing the fungal community. Our findings align with the results presented by Zhu et al. [10] and Ren et al. [33]. However, they diverge from Li et al. [17]’s conclusion which posits pH as the principal environmental determinant for soil fungi. A plausible explanation for this inconsistency might be that our study area represents an artificially established alpine grassland specifically for ecological restoration of mining zones. Given that the interaction mechanism between soil environmental factors and fungal communities in alpine regions is intricate, this study has predominantly approached from the angle of soil physicochemical properties. Future investigations should delve deeper, considering facets like plant diversity and its compositional attributes.

Our study area is situated at the world’s "third pole" on the Qinghai-Tibet Plateau, predominantly characterized by alpine mountain ecosystems. Here, aspect emerges as a primary topographic determinant and is an essential environmental factor when considering the ecological restoration and revegetation of mountain ecosystems in alpine regions. This research ventured into understanding the influence of aspect heterogeneity on fungal community composition and diversity, focusing solely on the homogeneity of artificially constructed alpine reconstituted soils for ecological restoration in the Muli mine. Going forward, heightened emphasis should be placed on discerning the mechanisms behind microbial differentiation between ecologically restored alpine meadows and neighboring untouched grasslands, as well as their evolution over time.

## Conclusion

This study revealed significant differences in the dominant phyla, classes, and species levels of the soil fungal community in artificially planted alpine grasslands across different aspects. When compared with the No aspect setting, the Shannon index of fungi decreased by 2.99%, 19.32%, 19.37%, and 10.56% for the East, South, West, and North aspects, respectively. Concurrently, the Chao1 index of fungi decreased by 2.44%, 35.50%, 42.15%, and 3.21% for these respective aspects. A total of 22 distinct fungal types were identified in the study area. Functional prediction of the fungal communities across different aspects, as analyzed by PICRUSt2, mainly centered around aerobic respiration I (cytochrome c) and aerobic respiration II (cytochrome c). Total P and total N emerged as primary influencers of the fungal community. In summary, aspect considerably altered the composition of fungal communities and decreased their diversity. These findings enhance our understanding of the construction mechanisms governing fungal communities at local scales in varied aspects.

## Supporting information

S1 File (RAR)

S2 File (XLSX)

S3 File (XLSX)
